# Effects of Different Amounts of Supplemental Selenium and Vitamin E on the Incidence of Retained Placenta, Selenium, Malondialdehyde, and Thyronines Status in Cows Treated with Prostaglandin F_2*α*_ for the Induction of Parturition

**DOI:** 10.1155/2013/867453

**Published:** 2013-11-07

**Authors:** Ivan B. Jovanović, Miljan Veličković, Dragan Vuković, Svetlana Milanović, Olivera Valčić, Dragan Gvozdić

**Affiliations:** ^1^Department for Physiology and Biochemistry, Faculty of Veterinary Medicine, University of Belgrade, Bulevar Oslobođenja 18, 11000 Belgrade, Serbia; ^2^Veterinary Clinic VELVET, Kaplarova 24a, 19350 Knjaževac, Serbia; ^3^Department for Reproduction, Faculty of Veterinary Medicine, University of Belgrade, Bulevar Oslobođenja 18, 11000 Belgrade, Serbia; ^4^Department for Pathophysiology, Faculty of Veterinary Medicine, University of Belgrade, Bulevar Oslobođenja 18, 11000 Belgrade, Serbia

## Abstract

The incidence of retained placenta (RP) in cows increases in cases of parturition induced by prostaglandin F_2*α*_. We analyzed the effects of different doses of supplemental selenium and vitamin E on the incidence of RP, blood selenium, plasma thyronines, and malondialdehyde concentration. Thirty-three clinically healthy, multiparous Holstein-Frisian cows were assigned to 3 groups and supplemented with a single intramuscular injection of sodium selenite (SS) and tocopherol acetate (TAc) between days 250 to 255 of gestation: control—unsupplemented; group A—10 mg SS + 400 mg TAc; group B—20 mg SS + 800 mg TAc. Parturition was induced using PGF_2*α*_ not before day 275 of gestation. The RP incidence was reduced from 66.7% in the control to 38.2 and 30.8% in groups A and B, respectively. Blood selenium and glutathione peroxidase activity in treated groups were significantly higher compared to control, with no significant difference between groups A and B. Plasma malondialdehyde in group B was significantly lower than that in control and group A, while thyronines levels were not affected. Comparison of RP and non-RP cows, independently of supplement treatment, revealed higher blood selenium and glutathione peroxidase activity and lower MDA and thyroxine in non-RP animals, while triiodothyronine level did not differ.

## 1. Introduction

Induced parturition in cows may have several technological and medical advantages over spontaneous delivery, such as avoiding unattended night-time calving, providing colostrum to the newborn in time, preventing dystocia caused by relative fetal oversize, dealing with fetal deformities, and hydroallantois [1]. Calving can be successfully induced by an intramuscular injection of prostaglandin F_2*α*_ (PGF_2*α*_). It acts on the *corpus luteum* to cause luteolysis and stops the production of progesterone. Time required from treatment to calf delivery ranges from 24 to 72 hours, on average 48 hours. However, the major problem arising from this procedure is a dramatic rise in the incidence of retained placenta (RP), up to 80% in some cases [2], although no other serious harmful effects on cows and calves have been reported [3].

Retained placenta (RP) is a reproductive disorder seen in cows and water buffalos. It is characterized by the inability of the animal to expel fetal membranes within 12 hours after parturition. Its incidence varies from 1.3 to 39.2% of overall parturitions, resulting in serious economic losses in the dairy industry throughout the world [4].

The etiology of RP is complex and not yet fully understood. In the majority of cases, it is directly caused by a disturbance of the prepartal loosening mechanism in the placentomes [5]. Dystocia and abortion/stillbirth increase the risk of RP. Furthermore, cows with RP are at high risk of developing metritis and silent heat, resulting in prolonged service period and low fertility rate [6, 7]. Various factors, such as age, breed, heredity, environment, hormonal status, nutrition, and immunity had been implied to contribute to the development of RP. However, no single factor was sufficient to provide a satisfactory explanation for the mechanism underlying the development of this disorder [8].

It is well established that innate and acquired immune defense mechanisms are lowest in the peripartum period due to metabolic strain during pregnancy, calving, and early lactation [9]. Two immune mechanisms had been proposed to explain the detachment of the fetal membranes during parturition. Both were based on the hypothesis that upon parturition, when blood supply to the placenta begins to cease, the placenta becomes a “foreign body” that the maternal immune system must recognize, attack, and expel. Gunnink [10] demonstrated that leukocytes were less able to recognize cotyledon tissue harvested from cows with RP compared non-RP. However, Kimura et al. [8] argued that 3–8 hours needed to expel fetal membranes was a too short time for lymphocytes to mount an effective rejection process; hence, they focused on the role of innate immunity mediated by neutrophils through their oxygen-dependent killing capabilities.

Pregnancy in dairy cows is considered to induce oxidative stress, which in turn can be a significant underlying factor to dysfunctional host immune and inflammatory responses that can increase the incidence of perinatal disorders during the transition period [11, 12]. Wischral et al. [13] proposed a pathway of RP development starting with an imbalance of the antioxidant capacity, followed by a decrease in estrogen production, resulting in decreased PGF_2*α*_ and accumulation of arachidonic and linoleic acids in the placental tissue. Therefore, it is necessary that the animal organism should be able to maintain its antioxidative/oxidative processes in balance during peripartal period.

Methods for quantifying oxidative stress mostly include direct or indirect measures of antioxidants and oxidants, or their metabolic products present in animal tissues. Malondialdehyde (MDA) is one of the several low-molecular weight end-products that arise from oxidative decomposition of polyunsaturated fatty acids. MDA readily reacts with thiobarbituric acid producing a red pigment that can be measured spectrophotometrically [14].

Selenium is well established as one of the key elements in the antioxidative defense of a living organism. In the active form, it is incorporated in glutathione peroxidases (GPx), a family of enzymes that use the reductive potential of glutathione (GSH) to convert hydroperoxides to corresponding alcohols [15]. According to Bernabucci et al. [16, 17], plasma GPx activity in cows raises several days prior to parturition and gradually declines afterwards, while in the erythrocytes, the activity remains constant. Vitamin E is the main chain breaking antioxidant residing within the cell membranes. However, in its reaction with the peroxyl radicals vitamin E produces hydroperoxides that are still toxic and must be removed [18] by GPx, hence the functional interdependence of selenium and vitamin E.

The first report on the relationship between Se and vitamin E status of cows and retention of placenta was published by Trinder et al. [19] drawing substantial attention to this subject. Experiments conducted by Jaskowski [20] and Wentink et al. [21] established a minimal selenium concentration in cow's blood plasma of 30 ng/mL, below which the incidence of RP would significantly rise, namely, from 5-6% to 20–22%. Preventive effects of different doses of Se and/or vitamin E against RP prepartally supplemented to cows, have been reported over the years by a number of authors [20–25].

All three known types of iodothyronine deiodinases (ID), enzymes which activate and/or inactivate thyronines through deiodination, contain Se in the form of selenocysteine in their catalytic sites. However, ID activities are not as tightly linked to Se status as the activity of GPx [26]. Therefore, serum thyroxine (T4) and triiodothyronine (T3) levels as well as T4/T3 ratio may only be slightly affected by marginal Se deficiency [27]. According to Nixon et al. [28] in dairy cows the stage of lactation and season of the year have a strong influence on changes in serum thyronines. Concentrations are the lowest in early lactation, during winter and early spring. Pethes et al. [29] reported that serums T4 and T3 concentrations in periparturient cows start to decrease 14 days prior to delivery and recover slowly within the first few weeks after parturition.

High incidence of retained placenta, together with a wide variability in the intervals between prostaglandin F_2*α*_ treatment and calf delivery, remains the primary limiting factor for the practical use of PGF_2*α*_ in the synchronization of parturition on dairy farms. The aim of this trial was to determine to which extent a preparturient supplementation with different doses of Se and vitamin E would influence the incidence of RP, as well as selenium, MDA, and thyronines status in cows treated with PGF_2*α*_ for the induction of parturition.

## 2. Material and Methods

### 2.1. Experimental Groups and Treatments

Thirty-three (33) Holstein-Frisian cows included in this investigation were randomly distributed to 3 groups and supplemented with sodium selenite (SS) and tocopherol acetate (TAc) as follows:

- *Control group* (*n* = 9) was not supplemented, and it served as a negative control;
- *group *A (*n* = 11) was supplemented 10 mg SS and 400 mg TAc;
- *group* B (*n* = 13) was supplemented 20 mg SS and 800 mg TAc.

The supplement was administered by a single intramuscular injection between days 250 and 255 of gestation; parturition was induced using a single intramuscular injection of PGF_2*α*_ (2 mL, 500 *μ*g of cloprostenol) not before day 275 of gestation; venous blood samples for analysis were taken 12 hours *postpartum*.

All animals were clinically healthy, multiparous, and single calve, with no previous record of retained placenta or other disorders.

### 2.2. Blood Se Concentration

Determination of selenium in whole blood samples of cows was carried out using atomic absorption spectrometry-hydride technique. Microwave digestion was used for sample preparation. Samples of whole blood (0.5 g) were accurately weighted using analytical balance Denver Instrument, model TB-215D (Denver Instruments, USA), transferred into Teflon microwave vessels, and digested with 8 mL of 69% nitric acid (Sigma-Aldrich, USA) and 2 mL of 30% hydrogen peroxide (Fluka Analytical, USA). Microwave oven (Milestone, Germany, model Touch control) was set to the following program: temperature ramp from ambient temperature at 180°C followed by 15 min of holding time and 20 min of cooling time. Digested samples were transferred to volumetric flasks and diluted using 5 M hydrochloric acid (Sigma-Aldrich, USA) to the final volume of 25 mL. Determination of selenium concentration was carried out using SolAAr, Series 4 spectrometer equipped with VP70 hydride module and EC90 electrical furnace for precise temperature control of the analytical cuvette (Thermo Electron, UK). Measuring absorption at 196 nm was performed after SeH_4_ formation in the hydride system with 5% NaBH_4_ (J. T. Baker, The Netherlands) and 0.6% NaOH (Merck, Germany). Stabilization and baseline delay times were 40 and 60 seconds, respectively, and reading time was 7 seconds in three replicates. Furnace was set to 900°C. 

Quantification of Se content was performed using five-point calibration curve (10–40 *μ*g/kg, including zero) of reference standard solutions (Merck, Germany). Quality control was achieved using blank samples fortified in 20 *μ*g/kg of Se and certified reference material (BCR 189). Good linearity was obtained from the calibration curve (*r* = 0.998), and the measured concentration of the reference material was in the range of the reference value.

### 2.3. Serum MDA Concentration

Serum MDA was measured using the spectrophotometric method introduced by Andreeva et al. [30]. All chemicals were obtained from Sigma-Aldrich. Briefly, a 3 mL of 0.1% orthophosphoric acid, 1 mL of 0.6% thiobarbituric acid, and 0.1 mL of 0.28% hydrated ferrous sulfate solution were added to 0.3 mL of serum. The reaction mixture was heated in boiling water for 60 minutes. The produced chromogen was extracted with n-butyl alcohol (4 mL). After centrifugation (2200 ×g, 10 minutes), the butanol layer was separated for spectrophotometric measurement at 535 nm. 

### 2.4. Blood Glutathione Peroxidase (GPx) Activity

Glutathione peroxidase activity was measured in whole blood samples by the coupled test described by Günzler et al. [31]. All chemicals were obtained from Sigma-Aldrich. Blood samples were hemolyzed using Drabkin's reagent (1.6 mM KCN, 1.2 mM K_2_Fe (CN)_6_, and 0.023 M NaHCO_3_). The GPx present in the samples reduces tertiary butyl hydroperoxide (TBH). Glutathione (GSH) as the donor of hydrogen becomes oxidized to GS-SG. In the second phase of this coupled reaction, GS-SG is reduced to GSH by NADPH and glutathione reductase (GR). Final concentrations of used reagents were 100 mM phosphate buffer (pH 7.4), 4 mM EDTA, 6 mM GSH, 0.375 IU/mL GR, 0.3 mM NADPH and 1.575 mM TBH. Low concentration of TBH (under 2.32 mM) as used in this method determines only the activity of Se-dependent GPx. The reduction of NADPH was followed for 3 min at 366 nm using a Cecil Ce2021 spectrophotometer (UK) with a Peltier thermostat unit. Absorbance (*A*) values were taken at 30 seconds intervals, and the results were expressed in microkatals per liter (*μ*kat/L).

### 2.5. Serum T3 and T4 Concentrations

Concentrations of T3 and T4 were measured in heparinized plasma samples using commercial standard RIA kits (INEP, Zemun). The assay is based on the competition between unlabelled T3 and T4 and a fixed quantity of ^125^l labeled T3 and T4 for a limited number of binding sites on T3 and T4 specific antibodies (bound to the tubes). Allowing a fixed amount of tracer and antibody to react with different amounts of unlabelled ligand, the amount of tracer bound by the antibody will be inversely proportional to the concentration of unlabelled ligand. Antigen-antibody complex is bound to the tubes, and the supernatant is then separated by decantation. Counting the radioactivity of the bound phase enables a standard curve to be constructed and samples to be quantified.

### 2.6. Statistical Analysis

Obtained results were arranged and analyzed from two aspects: Table 1 displays experimental results according to Se and vitamin E treatments as previously described, and Table 2 displays the results according to the absence/presence of retained placenta in cows, diagnosed 12 hours *postpartum*, independently from Se and vitamin E treatment. Data are presented as means ± SD. Analysis was performed using *MS Excel 2007* and *Graph Pad Prism 5* statistical software packages. The differences between experimental groups were analyzed using Student's *t*-test. A probability level *P* < 0.05 was considered statistically significant.

## 3. Results

The treatment of cows with 10 mg sodium selenite (SS) and 400 mg tocopherol acetate (TAc) reduced the incidence of retained placenta from 66.7% in the control group to 38.2% in group A, while treatment with 20 mg SS and 800 mg TAc further reduced RP to 30.8% in group B (Table 1). Blood selenium concentrations and GPx activities in both treated groups (A or B) were significantly higher compared to the control; however, there were no significant differences between groups A and B. Blood plasma MDA content was lowest in group B (3.95 ± 0.88 *μ*M), significantly lower than both control (5.70 ± 0.94 *μ*M) and group A (4.59 ± 1.20 *μ*M). Total plasma T4 levels were highest in group A (59.55 ± 13.00 nM) and lowest in group B (43.2 ± 16.2 nM), while mean T3 concentrations ranged from 1.1 ± 0.4 nM in group B to 1.7 ± 0.6 nM in control group.

Comparison between cows with and without the diagnosed RP (independently from SS and TAc treatment), revealed the following (Table 2): cows with RP had significantly lower blood Se content and GPx activity and higher plasma MDA concentrations compared to those free of RP. Plasma T3 did not differ significantly, while plasma T4 concentration was significantly higher in cows with RP.

## 4. Discussion

The primary aim of this study was to test the assumption that *prepartum* supplementation of cows with Se and vitamin E should exhibit the protective effect against the onset of retained placenta in cows with prostaglandin F_2*α*_ induced parturition in a similar fashion as in animals with spontaneous parturition. To our best knowledge, this is the first such attempt; therefore, the only available frame of reference for our data is the research done on animals with spontaneous labour.

In cows supplemented with Se and vitamin E, 20 days prior to parturition induced by PGF_2*α*_, the incidence of retained placenta was effectively halved, from 66.7% in the control group to 38.2% and 30.8% in groups A and B, respectively (Table 1). Similarly, Julien et al. [22] demonstrated that a single 20-day *prepartum* injection of 50 mg of sodium selenite and 680 IU of *α*-tocopherol acetate effectively reduced the incidence of RP from 51.2% in the control group of cows to 8.8% in the treated group. Selenium alone was at least as effective as a combination of selenium and vitamin E [23]. The higher the dose of selenium administered, the lower the incidence of RP was registered [24]. In all these experiments, the percentage of RP in herds was reduced to values close to zero, while in our trial the lowest value remained at 30.8%. Therefore, we estimate that further increase in Se dosage would not produce a linear but rather asymptotic decrease in RP incidence. One must always bear in mind that selenium is very toxic. The supplement dosage should be considered with great care, balancing between sufficient supply and avoiding toxicity. Marked discrepancies in dose/effect ratios described in the literature may be the result of different basal Se status of animals, which is highly dependable on naturally occurring Se levels in locally produced feedstuffs. According to investigations conducted by Jovanović et al. [32] in the region surrounding the experimental farm, Se content in cereals and hay ranged from 40 to 62 *μ*gSe/kg. These results are considered to be marginally deficient. Furthermore, as the retention of placenta is known to be a multifactorial disorder, our results demonstrate that certain effects of PGF_2*α*_ may not fall into the domain of oxidant-antioxidant balance in the body and cannot be overcome solely by the antioxidative actions of Se and vitamin E.

Blood selenium concentration is considered to be a good indicator of the long-term Se status in most animal species. Selenium concentration was significantly elevated (Table 1) in groups A (163 ± 28 ng/mL) and B (188 ± 30 ng/mL) supplemented with 10 mg and 20 mg sodium selenite, respectively, compared to unsupplemented control animals (129 ± 18 ng/mL). Cows suffering from retained placenta (Table 2) had significantly lower (*P* < 0.05) blood Se content (138 ± 40 ng/mL) compared to non-RP animals (176 ± 33 ng/mL). Kommisrud et al. [33] collected blood samples from 254 Se unsupplemented dairy herds in Norway and found that their blood Se concentrations ranged from 60 to 120 ng/g and proposed the content of 100–150 ngSe/g as a delimiting range below which a higher incidence of RP could be expected. Blood selenium concentrations measured in their study were slightly below the range of the values found in unsupplemented cows in our experiment. This reflects the fact that Norwegian crops are slightly more selenium deficient [34] than those in the northern part of Serbia [32]. However, both sets of data corroborate that the blood concentration of 150 ngSe/mL could represent the boundary between higher and lower probability for the onset of RP.

The activity of selenium-dependent glutathione peroxidase (GPx) in the blood of dairy cows from groups A and B (182 ± 32 and 186 ± 33 *μ*kat/L, resp.) was twice as high as in the control (91 ± 15 *μ*kat/L); however, activities did not significantly differ between groups A and B (Table 1). Blood GPx activity was significantly lower in cows with RP than in non-RP cows (Table 2). Comparable results were presented in studies conducted by Wischral et al. [13] and Kankofer et al. [35].

Plasma MDA concentrations were 5.70 ± 0.94 *μ*M in control, 4.59 ± 1.20 *μ*M in group A, and 3.95 ± 0.88 *μ*M in group B. MDA was significantly lower in treated animals (*P* < 0.05 in group A and *P* < 0.01 in group B) compared to control (Table 1). However, all of these values are generally substantially higher than those published by Wischral et al. [13], Erisir et al. [36], and Kankofer et al. [37]. We assume that high levels of MDA may be due to elevated oxidative stress caused by premature parturition induced by PGF_2*α*_. Animals with RP had significantly higher (*P* < 0.01) plasma MDA than non-RP cows (Table 2), which is in accordance with the findings of the above-mentioned authors.

Concentrations of plasma thyroid hormones, T4 and T3, in our experiment (Table 1) were below those published by Jovanović et al. [27] for 12 months old heifers fed Se-adequate feed but corresponded to the levels published by Pethes et al. [29] and Djoković et al. [38] for mature cows in late pregnancy. However, T4 and T3 did not apparently depend upon blood selenium supplementation of the animals. On the other hand, plasma T4 was significantly higher in cows with diagnosed RP, compared to non-RP (Table 2).

## 5. Conclusion

Our study demonstrated that in cows treated with single intramuscular injection of 20 mg sodium selenite and 800 mg tocopherol acetate three weeks *prepartum* there was a significant increase in plasma GPx activity and decrease in MDA concentration. Consequently, incidence of retained placenta in supplemented compared to nonsupplemented animals was reduced in half. This indicates that oxidative stress may be the principal, but not the only, cause of increased RP in animals with induced parturition. Further investigation is needed, using reasonably higher supplement doses of antioxidants, to show whether the incidence of RP can be brought to values closer to zero in a similar fashion as in cows with spontaneous calving.

## Figures and Tables

**Table 1:** The effects of three different SS and TAc supplemental treatments (mean ± SD) on the incidence of RP, Se, MDA, and thyroid status of cows 12 hours *postpartum*. Parturition was induced using prostaglandin F_2*α*_.

Group (number of animals)	Retained placenta (%)	Blood Se (ng/mL)	Blood GPx (µkat/L)	Plasma MDA (µM)	Plasma T3 (nM)	Plasma T4 (nM)
Control (*n* = 9)	66.7	129 ± 18	91 ± 15	5.70 ± 0.94	1.67 ± 0.26	49 ± 17
Group A (*n* = 11)	38.2	163 ± 28^**^	182 ± 32^***^	4.59 ± 1.20^*^	1.53 ± 0.40	60 ± 13^*^
Group B (*n* = 13)	30.8	188 ± 30^***^	186 ± 33^***^	3.95 ± 0.88^***^	1.14 ± 0.44^**^	43 ± 16

Compared to control (*t*-test): ^*^ *P* < 0.05; ^**^ *P* < 0.01; ^***^ *P* < 0.001.

**Table 2:** Selenium, MDA, and thyroid status of cows without (non-RP) and with retained placenta (RP) 12 hours *postpartum*, independently of SS and TAc supplemental treatment. Parturition was induced using prostaglandin F_2*α*_.

RP status (number of animals)	Blood Se (ng/mL)	Blood GPx (µkat/L)	Plasma MDA (µM)	Plasma T3 (nM)	Plasma T4 (nM)
Non-RP (*n* = 19)	176 ± 33	181 ± 34	4.68 ± 0.93	1.31 ± 0.41	42 ± 13
RP (*n* = 14)	138 ± 40^*^	133 ± 48^**^	5.32 ± 0.80^**^	1.37 ± 0.34	51 ± 11^**^

Between groups (*t*-test): ^*^ *P* < 0.05; ^**^ *P* < 0.01.

## List of Abbreviations

GPx: Glutathione peroxidase
ID: Iodothyronine deiodinase
MDA: Malondialdehyde
PGF_2*α*_: Prostaglandin F_2*α*_
RP: Retained placenta
SS: Sodium selenite
T3: Triiodothyronine
T4: Thyroxine
TAc: *α*-Tocopherol acetate (vitamin E).
